# 3-Oxo-3-(piperidin-1-yl)propane­nitrile

**DOI:** 10.1107/S1600536812035015

**Published:** 2012-08-15

**Authors:** Hoong-Kun Fun, Ching Kheng Quah, Hatem A. Abdel-Aziz, Hazem A. Ghabbour

**Affiliations:** a X-ray Crystallography Unit, School of Physics, Universiti Sains Malaysia, 11800 USM, Penang, Malaysia; b Department of Pharmaceutical Chemistry, College of Pharmacy, King Saud University, PO Box 2457, Riyadh 11451, Saudi Arabia

## Abstract

In the title compound, C_8_H_12_N_2_O, the piperidine ring exhibits a chair conformation and its least-squares plane (all atoms) makes a dihedral angle of 32.88 (12)° with the propane­nitrile unit (r.m.s. deviation = 0.001 Å). In the crystal, mol­ecules are linked by C—H⋯O hydrogen bonds, forming chains along [001].

## Related literature

For ring conformations, see: Cremer & Pople (1975 ▶). For background to piperidine derivatives, see: Andrews *et al.* (2008 ▶); Abdel-Aziz & Mekawey (2009 ▶); Abdel-Aziz *et al.* (2009 ▶, 2011 ▶). For the synthesis, see: Whitehead & Traverso (1955 ▶).
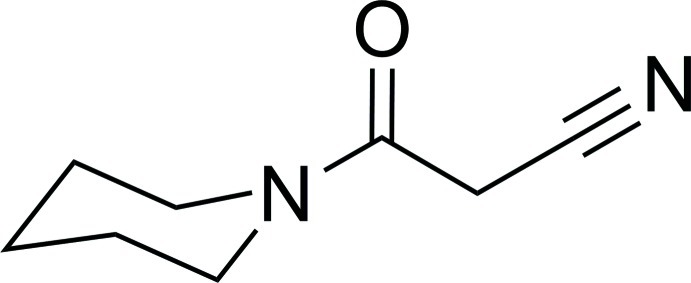


## Experimental

### 

#### Crystal data

C_8_H_12_N_2_O
*M* *_r_* = 152.20
Monoclinic, 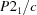

*a* = 9.7106 (2) Å
*b* = 8.9468 (2) Å
*c* = 9.8487 (2) Å
β = 101.425 (1)°
*V* = 838.69 (3) Å^3^
*Z* = 4
Cu *K*α radiation
μ = 0.66 mm^−1^
*T* = 296 K
0.70 × 0.62 × 0.39 mm

#### Data collection

Bruker SMART APEXII CCD diffractometer
Absorption correction: multi-scan (*SADABS*; Bruker, 2009 ▶) *T*_min_ = 0.656, *T*_max_ = 0.783
5110 measured reflections
1300 independent reflections
1222 reflections with *I* > 2σ(*I*)
*R*_int_ = 0.030

#### Refinement

*R*[*F* ^2^ > 2σ(*F* ^2^)] = 0.053
*wR*(*F* ^2^) = 0.128
*S* = 1.12
1300 reflections
101 parameters
H-atom parameters constrained
Δρ_max_ = 0.20 e Å^−3^
Δρ_min_ = −0.30 e Å^−3^

### 

Data collection: *APEX2* (Bruker, 2009 ▶); cell refinement: *SAINT* (Bruker, 2009 ▶); data reduction: *SAINT*; program(s) used to solve structure: *SHELXTL* (Sheldrick, 2008 ▶); program(s) used to refine structure: *SHELXTL*; molecular graphics: *SHELXTL*; software used to prepare material for publication: *SHELXTL* and *PLATON* (Spek, 2009 ▶).

## Supplementary Material

Crystal structure: contains datablock(s) global, I. DOI: 10.1107/S1600536812035015/hb6912sup1.cif

Structure factors: contains datablock(s) I. DOI: 10.1107/S1600536812035015/hb6912Isup2.hkl

Supplementary material file. DOI: 10.1107/S1600536812035015/hb6912Isup3.cml

Additional supplementary materials:  crystallographic information; 3D view; checkCIF report

## Figures and Tables

**Table 1:** Hydrogen-bond geometry (Å, °)

*D*—H⋯*A*	*D*—H	H⋯*A*	*D*⋯*A*	*D*—H⋯*A*
C7—H7*A*⋯O1^i^	0.97	2.23	3.1922 (17)	170

Symmetry code: (i) 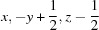.

**Fig. 1.**
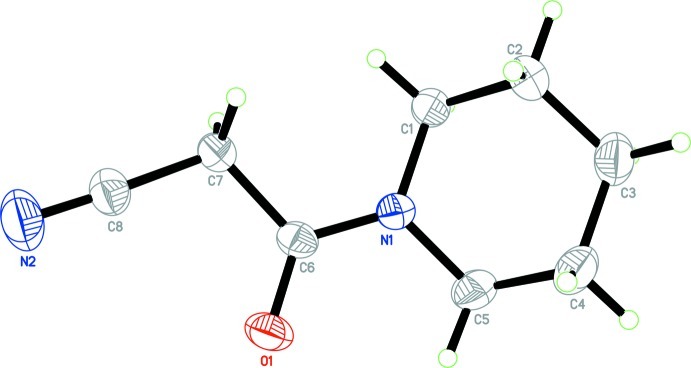
The molecular structure of the title compound showing 30% probability displacement ellipsoids for non-H atoms.

**Fig. 2.**
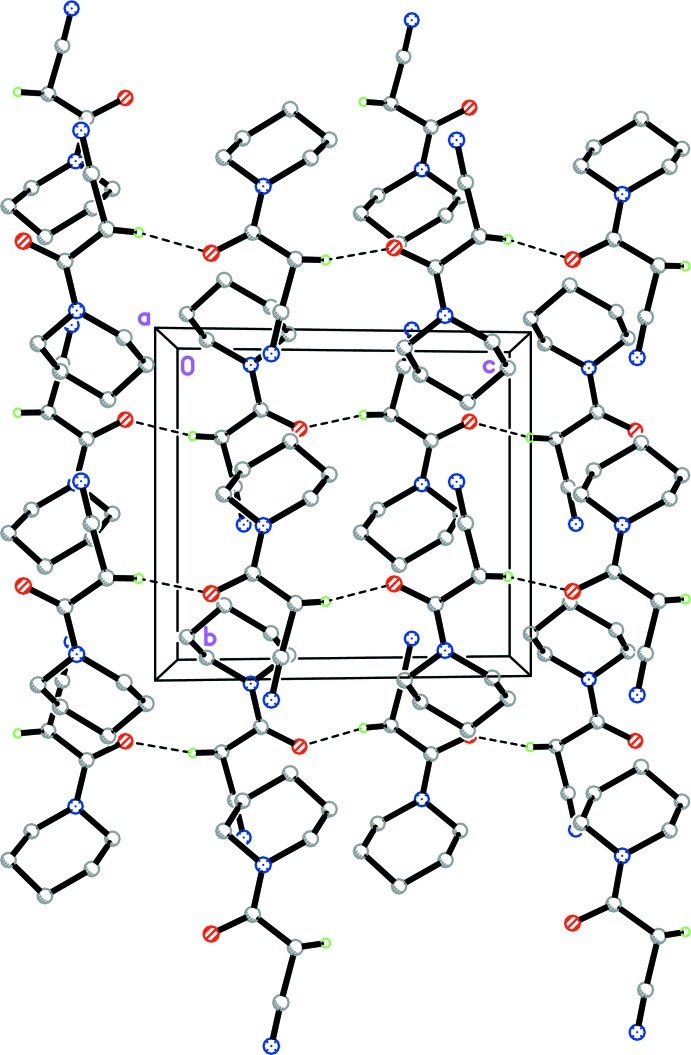
The crystal structure of the title compound, viewed along the a axis. H atoms not involved in hydrogen bonds (dashed lines) have been omitted for clarity.

## supplementary crystallographic information

### Comment

Piperidines are an important class of heterocycles found in numerous natural products and medicinal structures (Andrews *et al.*, 2008). In continuation of our interest in the chemistry of piperidines (Abdel-Aziz & Mekawey, 2009; Abdel-Aziz *et al.*, 2009, 2011), we report here the crystal structure of the title compound.

In the title molecule, Fig. 1, the piperidin-1-yl ring (N1/C1-C5) exhibits a chair conformation, puckering parameters (Cremer & Pople, 1975) Q = 0.5455 (18) Å; Θ = 1.84 (19)° and φ = 113 (6)Å, and its least square plane makes a dihedral angle of 32.88 (12)° with the propanenitrile unit (N2/C6-C8, *r.m.s.* deviation = 0.001 Å).

In the crystal (Fig.2), molecules are linked *via* C7–H7A···O1 hydrogen bonds (Table 1), forming chains along [001].

### Experimental

The title compound was prepared by the reaction of ethyl cyanoacetate with piperidine according to the reported method (Whitehead *et al.*, 1955). Colourless blocks were obtained by slowly evaporating an ethanol solution at room temperature.

### Refinement

All H atoms were positioned geometrically and refined using a riding model with C–H = 0.97 Å and *U*_iso_(H) = 1.2 *U*_eq_(C).

### Crystal data

**Table:**

C_8_H_12_N_2_O	*F*(000) = 328
*M**_r_* = 152.20	*D*_x_ = 1.205 Mg m^−^^3^
Monoclinic, *P*2_1_/*c*	Cu *K*α radiation, λ = 1.54178 Å
Hall symbol: -P 2ybc	Cell parameters from 2826 reflections
*a* = 9.7106 (2) Å	θ = 4.6–70.9°
*b* = 8.9468 (2) Å	µ = 0.66 mm^−^^1^
*c* = 9.8487 (2) Å	*T* = 296 K
β = 101.425 (1)°	Block, colourless
*V* = 838.69 (3) Å^3^	0.70 × 0.62 × 0.39 mm
*Z* = 4	

### Data collection

**Table:**

Bruker SMART APEXII CCD diffractometer	1300 independent reflections
Radiation source: fine-focus sealed tube	1222 reflections with *I* > 2σ(*I*)
Graphite monochromator	*R*_int_ = 0.030
φ and ω scans	θ_max_ = 63.0°, θ_min_ = 4.7°
Absorption correction: multi-scan (*SADABS*; Bruker, 2009)	*h* = −11→11
*T*_min_ = 0.656, *T*_max_ = 0.783	*k* = −7→10
5110 measured reflections	*l* = −11→11

### Refinement

**Table:**

Refinement on *F*^2^	Secondary atom site location: difference Fourier map
Least-squares matrix: full	Hydrogen site location: inferred from neighbouring sites
*R*[*F*^2^ > 2σ(*F*^2^)] = 0.053	H-atom parameters constrained
*wR*(*F*^2^) = 0.128	*w* = 1/[σ^2^(*F*_o_^2^) + (0.0759*P*)^2^ + 0.0948*P*] where *P* = (*F*_o_^2^ + 2*F*_c_^2^)/3
*S* = 1.12	(Δ/σ)_max_ = 0.001
1300 reflections	Δρ_max_ = 0.20 e Å^−^^3^
101 parameters	Δρ_min_ = −0.30 e Å^−^^3^
0 restraints	Extinction correction: *SHELXTL* (Sheldrick, 2008), Fc^*^=kFc[1+0.001xFc^2^λ^3^/sin(2θ)]^-1/4^
Primary atom site location: structure-invariant direct methods	Extinction coefficient: 0.82 (4)

### Special details

**Table:**

Geometry. All esds (except the esd in the dihedral angle between two l.s. planes) are estimated using the full covariance matrix. The cell esds are taken into account individually in the estimation of esds in distances, angles and torsion angles; correlations between esds in cell parameters are only used when they are defined by crystal symmetry. An approximate (isotropic) treatment of cell esds is used for estimating esds involving l.s. planes.
Refinement. Refinement of F^2^ against ALL reflections. The weighted R-factor wR and goodness of fit S are based on F^2^, conventional R-factors R are based on F, with F set to zero for negative F^2^. The threshold expression of F^2^ > 2sigma(F^2^) is used only for calculating R-factors(gt) etc. and is not relevant to the choice of reflections for refinement. R-factors based on F^2^ are statistically about twice as large as those based on F, and R- factors based on ALL data will be even larger.

### Fractional atomic coordinates and isotropic or equivalent isotropic displacement parameters (Å^2^)

**Table:**

	*x*	*y*	*z*	*U*_iso_*/*U*_eq_	
N1	0.24331 (12)	0.06319 (12)	0.22467 (11)	0.0473 (4)	
N2	0.08305 (19)	0.56540 (17)	0.19855 (19)	0.0850 (6)	
O1	0.24717 (12)	0.26280 (12)	0.36568 (9)	0.0599 (5)	
C1	0.19317 (15)	−0.01701 (16)	0.09524 (15)	0.0514 (5)	
H1A	0.1452	0.0519	0.0257	0.062*	
H1B	0.1266	−0.0934	0.1097	0.062*	
C2	0.31440 (19)	−0.08861 (19)	0.04488 (17)	0.0637 (5)	
H2A	0.3752	−0.0113	0.0205	0.076*	
H2B	0.2787	−0.1468	−0.0377	0.076*	
C3	0.39850 (19)	−0.1892 (2)	0.15449 (19)	0.0683 (6)	
H3A	0.3414	−0.2738	0.1705	0.082*	
H3B	0.4801	−0.2269	0.1224	0.082*	
C4	0.44535 (18)	−0.10440 (19)	0.28805 (19)	0.0673 (6)	
H4A	0.4908	−0.1729	0.3594	0.081*	
H4B	0.5136	−0.0293	0.2751	0.081*	
C5	0.32379 (19)	−0.0296 (2)	0.33513 (16)	0.0635 (5)	
H5A	0.2627	−0.1051	0.3621	0.076*	
H5B	0.3590	0.0322	0.4155	0.076*	
C6	0.20841 (13)	0.20271 (15)	0.25256 (12)	0.0425 (5)	
C7	0.11643 (16)	0.28844 (15)	0.13497 (14)	0.0491 (5)	
H7A	0.1588	0.2852	0.0537	0.059*	
H7B	0.0250	0.2410	0.1116	0.059*	
C8	0.09924 (16)	0.44374 (17)	0.17336 (16)	0.0558 (5)	

### Atomic displacement parameters (Å^2^)

**Table:**

	*U*^11^	*U*^22^	*U*^33^	*U*^12^	*U*^13^	*U*^23^
N1	0.0568 (7)	0.0441 (7)	0.0387 (7)	0.0043 (5)	0.0038 (5)	0.0000 (4)
N2	0.0915 (12)	0.0557 (10)	0.1066 (14)	0.0150 (8)	0.0165 (9)	−0.0146 (8)
O1	0.0771 (8)	0.0609 (8)	0.0407 (7)	−0.0035 (5)	0.0090 (5)	−0.0114 (4)
C1	0.0560 (8)	0.0436 (8)	0.0495 (8)	0.0026 (6)	−0.0017 (6)	−0.0064 (6)
C2	0.0770 (11)	0.0552 (10)	0.0575 (9)	0.0141 (7)	0.0097 (8)	−0.0108 (7)
C3	0.0657 (10)	0.0527 (10)	0.0837 (13)	0.0142 (7)	0.0077 (8)	−0.0032 (8)
C4	0.0628 (10)	0.0564 (10)	0.0736 (11)	0.0085 (7)	−0.0083 (8)	0.0097 (7)
C5	0.0808 (11)	0.0605 (10)	0.0448 (9)	0.0074 (7)	0.0018 (7)	0.0103 (6)
C6	0.0476 (7)	0.0455 (8)	0.0364 (7)	−0.0053 (5)	0.0133 (5)	−0.0027 (5)
C7	0.0615 (9)	0.0436 (9)	0.0428 (8)	0.0051 (6)	0.0115 (6)	−0.0030 (5)
C8	0.0597 (9)	0.0496 (10)	0.0600 (9)	0.0057 (6)	0.0167 (7)	−0.0037 (6)

### Geometric parameters (Å, º)

**Table:**

N1—C6	1.3361 (18)	C3—H3A	0.9700
N1—C1	1.4597 (16)	C3—H3B	0.9700
N1—C5	1.4650 (17)	C4—C5	1.508 (3)
N2—C8	1.134 (2)	C4—H4A	0.9700
O1—C6	1.2271 (16)	C4—H4B	0.9700
C1—C2	1.508 (2)	C5—H5A	0.9700
C1—H1A	0.9700	C5—H5B	0.9700
C1—H1B	0.9700	C6—C7	1.5224 (18)
C2—C3	1.514 (2)	C7—C8	1.458 (2)
C2—H2A	0.9700	C7—H7A	0.9700
C2—H2B	0.9700	C7—H7B	0.9700
C3—C4	1.508 (2)		
			
C6—N1—C1	125.76 (11)	C5—C4—H4A	109.2
C6—N1—C5	119.75 (11)	C3—C4—H4A	109.2
C1—N1—C5	113.99 (12)	C5—C4—H4B	109.2
N1—C1—C2	110.43 (11)	C3—C4—H4B	109.2
N1—C1—H1A	109.6	H4A—C4—H4B	107.9
C2—C1—H1A	109.6	N1—C5—C4	110.98 (13)
N1—C1—H1B	109.6	N1—C5—H5A	109.4
C2—C1—H1B	109.6	C4—C5—H5A	109.4
H1A—C1—H1B	108.1	N1—C5—H5B	109.4
C1—C2—C3	111.34 (14)	C4—C5—H5B	109.4
C1—C2—H2A	109.4	H5A—C5—H5B	108.0
C3—C2—H2A	109.4	O1—C6—N1	123.45 (12)
C1—C2—H2B	109.4	O1—C6—C7	119.88 (12)
C3—C2—H2B	109.4	N1—C6—C7	116.67 (11)
H2A—C2—H2B	108.0	C8—C7—C6	111.28 (11)
C4—C3—C2	110.49 (13)	C8—C7—H7A	109.4
C4—C3—H3A	109.6	C6—C7—H7A	109.4
C2—C3—H3A	109.6	C8—C7—H7B	109.4
C4—C3—H3B	109.6	C6—C7—H7B	109.4
C2—C3—H3B	109.6	H7A—C7—H7B	108.0
H3A—C3—H3B	108.1	N2—C8—C7	177.51 (18)
C5—C4—C3	111.85 (14)		
			
C6—N1—C1—C2	131.80 (14)	C3—C4—C5—N1	−53.1 (2)
C5—N1—C1—C2	−56.33 (17)	C1—N1—C6—O1	175.87 (13)
N1—C1—C2—C3	55.29 (18)	C5—N1—C6—O1	4.4 (2)
C1—C2—C3—C4	−54.4 (2)	C1—N1—C6—C7	−4.54 (19)
C2—C3—C4—C5	53.3 (2)	C5—N1—C6—C7	−175.99 (12)
C6—N1—C5—C4	−132.36 (14)	O1—C6—C7—C8	6.13 (18)
C1—N1—C5—C4	55.23 (18)	N1—C6—C7—C8	−173.48 (11)

### Hydrogen-bond geometry (Å, º)

**Table:**

*D*—H···*A*	*D*—H	H···*A*	*D*···*A*	*D*—H···*A*
C7—H7*A*···O1^i^	0.97	2.23	3.1922 (17)	170

Symmetry code: (i) *x*, −*y*+1/2, *z*−1/2.
